# Book Reviews

**Published:** 1806-03

**Authors:** 


### CRITICAL ANALYSIS

OF THE

### RECENT PUBLICATIONS

ON THE

### DIFFERENT BRANCHES OF PHYSIC, SURGERY, AND MEDICAL PHILOSOPHY.

---

*The Edinburgh Journal, No.* 5.

" Article 1.—*Observations on the Formation of the Human Ovum.* By Mr. John Burns, Lecturer on Midwifery, in Glasgow.

There are several ways of treating a subject. Most writers think it proper to begin with finding fault with their predecessors. When this is done as an apology for appearing before the public, it is expected that the opinions of others should be faithfully stated, with the arguments against them; after which, the reader is prepared to attend to the new doctrine from a conviction of the necessity to correct the old.

" It has been the opinion," says Mr. Burns, " of all the anatomists who have written on the subject of the gravid uterus, that the vascular part of the ovum, or the membrana decidua, is originally formed from lymph or extravasated blood. But the formation of organized animal matter is accomplished by a process

 very very different from the inspissation of lymph, or the production of new vessels through the medium of coagulated blood."

When we read of "all the anatomists who have written on the gravid uterus," we naturally turn our thoughts back as far as Aristotle, and wish to know what his opinion was on the subject. But when we afterwards meet with membrana decidua, it brings us on to the anatomist who first made use of that term, if he did not discover the membrane. Let us see how far Mr. Burns and his brother differ from those gentlemen, who are allowed to have thrown so much light on this important subject. After describing the increased vascular action of the uterus and its alteration in structure soon after impregnation, he proceeds,

"Next we find that, considerably within a month after impregnation, a number of vessels shoot out from that part of the membrane which lines the fundus uteri. These vessels have somewhat the appearance of the down which proceeds from putrid flesh. They are, however, of a different colour, thickly planted, and pretty closely united at their roots, but more separated at their extremities; so that, when the uterus is put in water, this part of it has a flocculent appearance. They never exceed the twelfth part of an inch in length. By degrees, still more of the uterine surface yields this vascular production, but it never is formed by the glandular part at the os uteri. This part secretes a jelly of a reddish colour, which is decomposed, and escapes very soon after death. The surface, however, immediately above the lacunæ yields a thin vascular production, which stretches across the uterus, and is evident as soon as the fundus has produced its down, whilst the intervening body of the uterus has as yet formed none.

"This vascular production forms the outer layer of the decidua. It has at first a striated appearance, from the direction of the vessels which go off at right angles to the uterine surface, which yields them. The striæ are alternately white and red, or black, owing to the empty arteries and distended veins; but if the uterus has been injected, then they are altogether coloured. This striated structure of the outer layer of the decidua seems, in the course of gestation, to be destroyed chiefly by pressure; for, by the end of the second or the beginning of the third month, it has more of a laminated appearance.

"I now go on to observe, that, almost immediately after this efflorescence of vessels proceeds from the uterus, (forming the outer layer of the decidua, or what may, at this period, be called the decidua striata) a second set of vessels is produced from the extremities of the first. These vessels are very different from the former, which are short, straight, and parallel to each other. These, on the contrary, are more extended, intermix and ramify together, so as to form an irregular tissue or sheet of vascular substance, the fibres or vessels of which assume a direction at right angles to the shag or primary vessels which formed them. This direction is very naturally given by the weight of the vessels themselves, which makes makes them hang down, or point toward the os uteri. This production constitutes the internal layer of the decidua,—the decidua interna. It is much more irregular than the outer layer; many ragged flocculent processes hang down in the cavity of the uterus, and extend toward the cervix; so that this layer presently appears to consist of a number of torn floating membranes, like portions of spiders' web, hanging pendulous in the uterus; but nearer inspection shows that there has been no laceration, the margin being smooth and well defined. These I would call the processes of the decidua interna."

Now, we who have not sagacity enough to *determine* how the decidua is formed, and are indifferent whether one part of it is called membrana decidua, *reflexa* or *interna*, are a little at a loss to know whether Mr. Burns intends to instruct us by telling us that "a number of vessels shoot out" in certain directions, or that the "uterine surface *yields* this vascular production," or that it "is *formed*." Whether it is *formed*, or *yielded*, or *shot out*, it does not become us to explain, and we are ready to confess our ignorance. But from those who assure us that the formation of organized animal matter is accomplished by a process very different from [the *inspissation of lymph*, or] the production of new vessels through the medium of coagulated blood, we expected to learn something more than Mr. Burns has taught us. We do not want proofs of coagulated blood acquiring blood-vessels from neighbouring parts, nor are we certain that under certain circumstances the formation of vessels in the coagulum itself may not be within the resources of the constitution to preserve and restore itself. In cases of hæmorrhage and of parts divided by the interposition of coagulated blood, such appearances are discovered. In forming the capsules of abscesses, we find coagulable lymph organized, and in the exterior coat of hydatids, which enables them to adhere to solid parts; in worms enclosed in solid parts we find a kind of nidus formed of coagulated lymph. It is not our intention to consider the formation of the decidua as the mere consequence of such a process; it is enough that such a process is consistent with the operations of nature; when any thing more satisfactory is pointed out, we shall thankfully attend to it.

" Article 2.—*On the Degree to which Exercise should be carried in the Cure of some Varieties of Dyspepsia;* by A. B. Faulkner, M. D. London.

The author found in one case, which had proved particularly obstinate after every attention to diet, medicines, and exercise, that the latter was only useful when it produced perspiration, and was used whilst the stomach was nearly empty. This induced him to apply the same mode of treatment to several other cases, and his success did not disappoint him.

" Article

"Article 3.—*Letter on the Use of sulphurated Hydrogen in stomachic Complaints;* by Mr. WILLIAM FORBES, Surgeon, Peterhead."

THIS paper contains some very useful observations in pointing out the advantages of a remedy but little attended to.

"Article 4.—*Account of Dr. Rousseau's Experiments on Cutaneous Absorption;* by J. G. STOCK, M. D. Bristol."

IN this paper Dr. Stock gives an abstract of Dr. Rousseau's inaugural Treatise, delivered at the University of Pensylvania in the year 1800. The following are the Doctor's opinions, which are afterwards, in part, supported by very well conducted experiments.

"Were the mercury absorbed in friction, it would certainly, after a long use, be found in some parts of the body; but I have never been able to discover any marks of the presence of mercury in the urine, milk, or other fluids of animals, upon whom it had not been spared."

"From several experiments, which are not yet sufficient to enable me to say any thing positive upon this subject, I am induced to believe, that mercury being a substance volatile enough to be capable of rising and diffusing emanations by a moderate degree of heat, (as has been proved by Mr. Achard's experiments,*) it may, with the assistance of the heat of the body of those using the frictions, be raised in very minute particles, as musk, camphor, spirit of turpentine, garlic, and others, and absorbed in the same manner as these volatile substances are. May it not be supposed also, that, during the frictions, some of the mercury is, by the action of the air, assisted by the heat of the body, oxyded, that it afterwards parts by degrees with its oxygen, which carries along with it to the lungs (which, I am to prove, are the organs performing absorption) some parts of the mercury, which is with difficulty separated from it? †

"He goes on to observe, that, 'From all the facts and observations

---

"* This gentleman, having left a dish containing twenty pounds of mercury over a furnace which was daily heated, after some days experienced a salivation, as did two other persons who had not quitted the room. This heat he estimates to be about 72° of Fahrenheit.—*Journal de Physique, Octobre*, 1782.

"One of the Professors of this University (Pennsylvania) experienced a similar effect in his own person, in consequence of being repeatedly in a close room with a patient under a profuse salivation from mercurial frictions. And two others have noticed a similar effect to have been produced upon persons who resided in a ward in the Pennsylvania Hospital, where mercurial friction was administered to several patients.

"† Oxygenous gas, obtained from the mercurial oxides, almost always holds a small quantity of mercury in solution. I have been a witness to its having produced a speedy salivation on two persons who used it for disorders of the lungs.—*Chaptal's Chemistry*.

ations that he has been able to collect from others, as well as from his own observations and inquiries, he had never been able to determine the absorption of any substance by the lymphatics; on the contrary, that every experiment which he had made, with the view to come at such discovery, had convinced him that the lungs are the only organs by which absorption is performed. He admits that various active substances may be introduced *under* the cuticle; but this, he says, " cannot certainly be called absorption. In such cases the virus is carried on by the circulating fluid with which it has been mixed. Anatomists dissect putrid bodies with perfect safety, and no noxious matter is absorbed by their hands, as long as the cuticle is not injured. Would not surgeons be exposed every day to the most dangerous consequences, if the matter of venereal and gangrenous ulcers could be absorbed by the entire skin? Even the venom of the viper may be laid upon the skin without any accident ensuing."

Dr. R's experiments were confined to turpentine. By exposing his mouth or nose for ever so short a time, he always produced the violaceous smell in his urine, but if he made a communication between the organs of respiration and the open air, whilst every part of the external skin was exposed to the effluvia, and even to the application of turpentine, no such consequence followed.

The experiments appear very satisfactory, and the inductions very rational, but some important facts seem to militate against the latter, or the author seems to wish to infer more from the former than is necessary. The manner in which mercury finds its way into the system we pretend not to ascertain; but it is certainly possible that a substance so infinitely divisable may be forced by friction through the pores of the skin and afterwards absorbed.

The author's analogy from animal poisons is unsatisfactory. Though the cuticle is for the most part a security against the poison of the viper, yet hitherto, we believe, few experiments have been made of friction in the manner mercury is applied. As to the morbid poisons, there is no doubt that both the venereal and variolous may produce their effect though the cuticle remains sound. Even that mild disease, the cow-pox, has been found to produce its proper irritation from friction, when no rupture of the cuticle has been suspected. We would not be thought to undervalue Dr. Rousseau's experiments on this account. Without doubt many substances are found to produce violent effects where the cuticle is broken, which are innocent where that membrane is entire. The mercurial salts particularly seem to produce no effect on the sound skin; but many cases are on record, and we could from our own knowledge produce others, in which salivation has followed the application of them to open ulcers or slighter interruptions of the common covering. Perhaps a sight of the whole of Dr. R's paper may explain his inferences further; but at present they are not completely satisfactory to us.

" Article

"Article 5.—*Analysis of a Stearoid Tumour;* by JOHN BOSTOCK, M. D. Liverpool.

THIS is another instance of the great diligence of Dr. Bostock, in one of the most important subjects, the perfect investigation of which is absolutely necessary in our future progress towards rational physiology and pathology. We shall not undertake to shorten the paper, which, like every thing that comes from its author, contains nothing superfluous. Great as its value is, it can only serve as a record, which those who are engaged in similar enquiries will not fail to consult in its orignal form.

"Article 6.—*History of a Case of Diabetes Mellitus successfully treated;* by HENRY FRASER, M.D. London.

THIS case is not sufficiently uniform in its symptoms to ascertain its reality, nor does it appear that any analysis of the urine was undertaken. The paper contains some handsome compliments to Dr. Willan and the late Dr. Woodville. The last is considered as a tribute of gratitude from a pupil to his teacher.

"Article 7.—*An Account of a Fœtus found in the Abdomen of a Woman eighty-three Years of Age;* by J. GRIVEL, Accoucheur in the Hospital of Villeneuve at Dresden, as communicated in the following Letter to Dr. Pearson, Leicester Square.

"Sir,

"I take the liberty of addressing to you a few lines, to desire you to accept the two drawings which I have sent, done as faithfully as my poor talent in the art enables me. You must know that we are in no want of bodies at Dresden, having, besides those of the hospital, all those of murderers and suicides. We lately received the body of Maria Walther, born at Meissen, widow of a Prussian hussar, who had attended in several campaigns in the capacity of a sutler to Frederic II. King of Prussia's army. She has been kept in the Lazarus Hospital since 1794 through charity, on account of her great age only, being in perfect health, and having never complained of any pain in the belly. She talked of nothing but of the wars in her former days, and assisted as a nurse to the sick in the Lazarus Hospital till within two hours of her death. On opening her body, the young surgeon who was employed was surprised to find a child in an indurated state, and blended, as it were, with the intestines. It appeared as represented in the drawing, No. 1. A more distinct view appeared after washing the parts, and as represented in the drawing. No. 2. We consider this as a most rare case, the woman being *eighty-three years of age*, as was proved by an extract of her baptism. I wished much to have purchased for you the original; but I was unable, it being reserved for the cabinet of the Elector. I have the the honour of recommending myself to your protection, and of being your very humble servant,

"J. Grivel.

"P. S. We have a girl (*une fille nine**) now in the Hospital who is much deformed, and far advanced in pregnancy. Her pelvis is not two inches, English, wide. We shall be obliged to perform the Cæsarian operation. This is the second of this description which we have had at the Hospital within these eighteen months.

"*Dresden, June* 28, 1805."

*Remarks by Dr. Pearson.*

"1. Although the above statement by no means contains a relation of many particulars, which a well-informed person would desire, and although, from the want of a more full account, it will be impossible to make many inferences, which otherwise could have been established, there is no just reason to question the fact that a fœtus was found in the abdomen as described.

"2. I have heard, within the last six months, of three similar cases, viz. one which fell under the observation of a very experienced physician of the first reputation in Gloucestershire. I have been favoured with a sight of the drawings of this case, and I know it is destined for publication: it will, there is no doubt, be given very correctly, and will contain a minute history of all the particulars belonging to so extraordinary an occurrence. A second instance of the same sort fell under the observation of a physician at Glasgow; and, as I was informed by a young physician, my pupil, in Edinburgh, it was so similar to the Gloucestershire case, that, while I was relating it in a lecture the last winter, he supposed it was the same as that with which he was acquainted at Glasgow. A third instance was communicated to me yesterday at Woolwich by Dr. Rollo: it occurred in Ireland, of which an account had been seen a few days ago in a letter.

"3. Even if I had leisure, I should not be disposed to make a number of remarks, and endeavour to explain the nature of these occurrences, there being so many professional men better qualified.

"* *Nine* is not a classical word in the French language, in which the letter was written, but we understand it to be a cant term used in some hospitals on the Continent, for those cases of deformity in the female, in which parturition must be difficult, if not impossible.—*Editors.*"

As this is a matter of some importance, we recommend a more particular enquiry into the true import of the word, in which we shall not fail to add our diligence. At present we only take the liberty to remark, that *naine* is a legitimate word in French, meaning only a female dwarf, and as it is applied according to the idiom of the language to both sexes, cannot refer to parturition. It is possible, though not likely, that the uncertainty of manuscript reading may have caused this doubt; but whether this is the case or not, most English readers will wish to be satisfied on the subject.

qualified. However, one question I shall venture to propose, in order to obtain from others a satisfactory explanation. What are the powers or circumstances which prevent the fœtus from undergoing the compositions and decompositions usual in the process of putrefaction? Three different reasons may be assigned.

"1. That the fœtus is in a dead state; but the exclusion of air containing oxyen gas is the circumstance which prevents putrefaction.

"2. That the fœtus is in a dead state; but the agency of living surfaces in contact with it, counteracts the putrefactive process.

"3. That the vitality of the fœtus is not extinguished, although the mode of agency of the principle of life is different from what is usual.

"The first supposition does not appear to me satisfactory, because air certainly does permeate through every part of the animal economy; and it is common for dead animal matter to become putrid in many cavities to which air has not a more free access than into the cavity of the abdomen. Also out of the animal body, we know that animal fluids, kept in glass vessels quite full, and hermetically sealed, afford new compositions by the play of chemical attraction.

"The second supposition may, perhaps, be thought not altogether improbable, being supported by analogy. So the fluid in dropsies, and in many other diseases, remains for months, and even years, in cavities, without alteration in its composition, seemingly owing to its being in contact with living matter. Generally, however, when lifeless animal matter is exposed to living surfaces, and confined so as to be much excluded from the air, it is either absorbed, or it excites the formation of pus, or it putrefies. The late Mr. Pierce Smith, my most ingenious pupil, wrote a paper, which was read at the Royal Society about the year 1795, containing many experiments to show, that when flesh of various sorts was inserted into the cavities of wounds purposely inflicted, it was absorbed, if in a certain quantity, and if the animal was in vigorous health; but if the quantity was too great, then purulent matter was formed, and, in some cases, the flesh introduced became putrid.

"The third supposition, viz. that vitality remains, appears most reasonable, because certain actions go on, which produce changes only known in living bodies; such as the ossification of a great part of the fœtus; the process of induration; the thickening of parts, and perhaps adhesion, from the communication of new vessels between the confined extraneous body, and the cavity in which it is contained."

We heartily agree with Dr. Pearson in the probability of his correspondent's case. By the questions proposed, "what are the powers or circumstances which prevent the fœtus from undergoing the compositions and decompositions usual in the process of putrefaction?" We apprehend our author means only to enquire what what laws have hitherto been discovered in dead or living animal matter, which may account for the fœtus continuing so long within the cavity of the abdomen, without putrefaction? We are quite as ready as he is to give up his first proposition, though we should have wished to be informed further of the permeability of all the living parts of the animal economy to air in an elastic state, if such is the meaning of our author, and also of those cavities in which it is common for dead animal matter to become putrid?

The second proposition, like many others, which are gradually becoming current without any recollection of the source from which we derive it, was long since taught by Mr. Hunter; nor do we find it contradicted by the experiments of Dr. Pearson's ingenious pupil. That animal matter, when inserted in sections made thro' the integuments, and parts below, of living animals, should be followed by such consequences, is perfectly analagous to all that is to be met with in Mr. Hunter's treatise on the blood and inflammation. That it should be absorbed, as extraneous, is a part of his doctrine of the absorption of solid parts; that it should stimulate under other circumstances to suppuration, is only a part of what we there meet with, as the means of removing extraneous bodies. If in some instances putrefaction followed, this might easily be accounted for by the manner which the foreign flesh was introduced, and the uncertainty whether it was instantly closed by the adhesive inflammation taking place in the living animal.

Dr. Pearson's last supposition, though seemingly supported by something like a climax, is to us unintelligible. Can the author mean, that a fœtus, which could not have remained in the abdomen according to the common laws of the human functions much less than forty years, still retained its life? As to the actions alluded to, they seem perfectly analogous to what are observed under other circumstances. For a time, part of the dead fœtus would be absorbed by the vessels of the mother in contact with it; and if this process ceased, what was left might be incased, and its interstitial substance filled with calculous matter, as we find happens to other extraneous bodies. The following Paper shows that this process may be carried so far, as even to affect the living part in which this altered action is set up.

" Article 8.—*Remarkable Case of an Ossified Fœtus and Uterus in a Woman sixty years of age.* By JOHN CALDWELL, M. D. Ireland, Londonderry."

This case was attended with a number of interesting particulars. The subject had many symptoms of labour under which she died. The following is the account of the dissection.

" She died about eight next morning, after a night apparently easy, and sleep frequently repeated. Her body being opened by Mr. School, jun. her intestines were, in some places, marked with black spots of incipient mortification, dilated with flatulency.

Her

Her liver was extremely small, but not diseased. The uterine tumour was large and irregular, with its small end to the neck of the uterus. It was evidently a child ossified, and connected with the uterus, the greater part of which was ossified. The head, with the face well marked, and one arm coiled, but obviously perceptible, formed the larger end of the tumour felt above the pelvis; and the part opened by scissars, and considered the head, during the attempts to deliver, was the trunk of the body, and lower extremities compressed. The whole tumour resembled a deformed bust, having the head, neck, and trunk. There was no unnatural adhesion of the uterus, by previous inflammation, to the surrounding parts of the pelvis or abdomen. The child and uterus, ossified together, were sent to Dr. Clarke, professor of midwifery in Dublin."

" Article 9. *Essay on Erythema Mercuriale, or that Eruption which sometimes occurs from the Use of Mercury.* By JOHN MAC MULLIN, M. D."

We had prepared a long critique on this paper, but on re-considering the subject, shall content ourselves with announcing the article, observing only, that too much attention cannot be paid to every symptom which occurs under the exhibition of so important a remedy as mercury.

" Article 10. *Second Essay on the Analysis of Animal Fluids.* By JOHN BOSTOCK, M. D. Liverpool."

In this valuable paper the ingenious author pursues his former enquiry with the same diligence and acumen as before. Having ascertained the charcters of albumen, jelly, and mucus, and pointed out the tests by which they may be ascertained with ease and precision; his object is now to propose a method of analysis of the compound fluids, of which these substances form a principal part.

Though heat was found an accurate test of the presence of albumen, yet it afforded no means of separating it from water, or any other substance. The principal test employed was oxymuriate of mercury, by which he was enabled so ascertain the quantity of albumen contained in any compound animal fluid.

The uncoagulable part of the albumen ovi, was found generally to consist of about $\frac{1}{4}$ part of the weight of the solid contents. A solution of this substance in 100 times its weight of water, was only affected by aqua lithargyri acetati, a single drop of which, produced a copious precipitate, which, from the effects of evaporation, appeared to be pure mucus.

The author further remarks, that in all his experiments on albumen, he observed a remarkable backwardness in this substance to putrefy, and when that process had taken place, the odour emitted resembled pus; whereas, the putrid mucilage discharged its usual nauseous smell.

The

The saline ingredients were so minute, that the author, with his usual candour, acknowledges himself unable to determine their nature.

The method of ascertaining the quantity of jelly in any compound fluid was found more easy. By mixing a solution of isinglass with a certain quantity of infusion of galls of a determined strength, the comparative quantity of the two may be ascertained from the precipitate.

In determining the proportion of mucus, in any given substance, Dr. B. found so many difficulties, that he was, at last, under the necessity of ascertaining the quantity of albumen and jelly, and of considering the remainder as mucus. But as some portion of salts always exists in all animal matter, further elucidation is, of course, requisite.

Having made this progress in the three most common components of the fluid parts of animal matter, our author's next object was to apply this analysis of animal fluids to the actual examination of some substances. His first set of experiments was on the fluid discharged from a tumour forming the disease usually called Spina Bifida, which he found to consist of

| | | | |
|---|---|---|---|
| " Water - - - | 97 | 8 | |
| Muriate of soda | 1 | 0 | |
| Albumen - - - | 0 | 5 | |
| Mucus - - - | 0 | 5 | These proportions are in some measure conjectural |
| Jelly - - - - | 0 | 2 | |
| Lime - - - - | | | A very minute quantity |
| | 100 | 0 | |

The next analysis was on the liquor pericardii, which was reduced to the following substances.

| | | | |
|---|---|---|---|
| " Water - - - | 92 | 0 | |
| Albumen - - | 5 | 5 | |
| Mucus - - - | 2 | 0 | The proportion of these substances is somewhat conjectural |
| Muriate of soda | 0 | 5 | |
| | 100 | 0 | |

The following is Dr. Bostock's account of his analysis of saliva, and his recapitulation of what has been done by others.

" The next analysis that I attempted was that of the saliva; this fluid, in its natural state, is mixed with such variable proportions of water, that it is almost impossible to fix any standard which can be considered even as the average quantity. It is, however, convenient, in observing the effects of reagents upon it, to have it in a more diluted state than it usually occurs; and I accordingly united it by rubbing it in a mortar, with a quantity of distilled water, until, by evaporation, 100 grains of the mixture were found to contain two grains of solid residuum. Upon this mixture the following experiments were performed.

 " 1. The

"1. The fluid was still opake, and there was an appearance as if some flocculent matter were suspended in it.

"2. No effect seemed to be produced by exposing it to the boiling temperature.

"3. When the oxymuriate of mercury was added, no immediate visible effect was produced; but, after some hours, a light flocculent coagulum separated and fell to the bottom, leaving the fluid nearly transparent.

"4. A portion of the fluid, left for a few days without addition, gradually suffered a quantity of matter to separate from it, as in No. 3; but the separation was less complete, and it was much longer in taking place.

"5. A quantity of the fluid being passed through a filter of bibulous paper, was rendered perfectly transparent.

"6. The oxymuriate of mercury being added to a quantity of No. 5, a very slight precipitate only was produced after some time.

"7. The addition of the infusion of galls to No. 1, caused a precipitation of white flakes; but, after filtration, the galls produced no effect.

"8. The filtered fluid, No. 5, produced a copious precipitate with the aqua lithargyri acetati.

"9. It also produced a considerable precipitate with the nitromuriate of tin.

"10. And with the nitrate of silver.

"11. Equal weights of the fluid, before and after filtration, were separately evaporated, and the amount of the residuum being ascertained, the quantities left were to each other nearly as 12 to 8.

"12. The diluted saliva, both before and after filtration, slightly reddened a paper stained with litmus.

"From these experiments we may draw the following conclusions. From No. 3, it would seem that the fluid contains albumen; but it appears from Nos, 1, 2, 4, 5, and 6, that the albumen is not soluble in water, but in that state in which it is found after coagulation. From this we learn, that it constitutes only 0.8 of a grain in 100 grains of the fluid. From No. 7, we learn that there is no jelly; from 8, 9, and 10, that there is a quantity of mucus and muriate of soda; and, from comparing these with each other, we are led to conclude, that the last substance exists only in small quantity."

This Number contains, as the first article of Medical Biography, an account of the life and writings of the late Dr. Currie, of Liverpool. We conceived there could be only one opinion of this truly respectable and amiable character, which made us feel the more when we met with the following passages.

"The reports on the effects of cold water, as a remedy in fever, were written to recommend a practice, of which Dr. Currie may in strict justice be considered as the author. It is true, that in in the records of the ancients, and in the writings of the moderns, we occasionally meet with an account of this practice. But those who employed it did so empirically, or without any fixed principles. It remained for Dr. Currie to ascertain its mode of action, to trace its consequences, and to lay down rules for its application. This he has effected in the most masterly manner. Had Currie not lived, or had he forborne to write, this gigantic remedy might still have lurked in obscurity, or might have been productive of infinite mischief, in place of universal advantage. At present it is morally impossible that any one who has read the medical reports can undesignedly misapply this remedy.

"To arrest the progress of fever, to circumscribe the range of death, to exterminate disease, and to nip contagion in the bud, though present to our dreams of felicity, would seem denied to our waking hopes. As far, however, as these objects will admit of attainment, we may expect them from the remedy which Dr. Currie has recommended to our notice. He has taught us to divest the jail-fever, the small-pox, and the scarlet-fever, of most of their terrors."

No man can be more grateful for the series of experiments contained in the reports than ourselves. But we do not on that account, wish to undervalue the labours of others; and if the formidable diseases above alluded, have lost most of their terrors, we suspect, it can only be among those who see the least of them.

A fourth paper of The Enquirer follows; on the chief diagnostic symptoms of hydrocephalus internus, and of the diseases which arise from worms in the intestinal canal.

Among the medical intelligence is the second report of the board of health.—Some doubts relative to Pacchioni's discovery.—Premiums proposed by the University of Wilna, relative to diabetes, and the probability of saccharine secretions in various parts of animals and plants.

---

*A Reply to the Anti-vaccinists.* By James Moore, Member of the Royal College of Surgeons, in London, 8vo.: London, 1806.

This is one of the liveliest, and at the same time, best arranged pamphlets that we have met with on the late controversy. It embraces the question historically, scientifically, and practically; dwells with minuteness, but without prolixity, on every part, and shews with great clearness, the favourable result that must follow such an inquiry into this valuable discovery. From this account our readers will readily perceive the impossibility of abridging such a performance, we shall therefore only offer a few extracts, to show that we have not overvalued the whole. As members of the faculty, we cannot but transcribe the introductory compliment, in which the fraternity is so handsomely treated:

"Could any class of human beings live in peace, it might be expected of those brought up to the Church and to Medicine.

 But

But ecclesiastics can seldom instil into mankind the doctrine of love to each other, without mutual rancour; and physicians as rarely inculcate acts of humanity, without snarling at their brethren. It is strange, yet true, that seamen and soldiers, whose duty it is to wound and destroy, are hardly more given to quarrel, than churchmen and physicians, whose avocation it is to relieve and to save. That vaccination should occasion contention, was a thing of course; but this has been carried to unexpected lengths; for both those who approve, and those who disapprove of vaccination, have accused each other of murdering their patients. It is to be regretted that they are not more prudent, for the public may give implicit credit to both.

" It must be owned indeed, that on this occasion, there was superadded to the general tendency of doctors to differ, a particular motive which rarely fails of having that effect upon all mankind. Small-pox was the source of no inconsiderable portion of the income of every medical practitioner; in so much that neither physicians nor surgeons would abandon this disease to the management of the other. The physician claimed it as a contagious fever, and therefore a medical case; but as the surgeon was the inoculator, he did not choose to relinquish the profits of the subsequent treatment. While each was eager for the whole, it was hardly to be expected, that a plan to take it from both, would be kindly received by either.

" Jenner's discovery was a touchstone, to detect what proportion of selfishness alloyed the human heart. It was calculated to make known, whether the scenes of misery, which medical men are compelled to witness, blunt their feelings. The result has certainly reflected distinguished honour on the faculty; for the plan to exterminate the small-pox, has been zealously adopted by the medical men of every part of the world which it has reached. There are, however, and I acknowledge it with reluctance; a few practitioners, who must be excluded from participating in the praise thus acquired; but it would be unjust to censure them, merely for opposing vaccination. It is more liberal to believe that their opposition is founded upon a conviction of its inefficacy.

" A difference of opinion is so natural, that the discovery had hardly been promulgated, when a controversy arose between those who admired it. Some of Dr. Jenner's followers attempted to pilfer the laurel crown which he had gained. They insinuated, that though he had accidentally fallen upon the discovery, they were alone capable of bringing it to perfection. They pretended to have studied the subject more profoundly, and to understand it better. Some curious experiments were then made public, shewing the effects of inoculating children both with small-pox matter and the vaccine fluid, under a variety of circumstances; the two were even mixed together, and this mixture was made use of for inoculation. All this is thought extremely innocent by some philosophers; but had any mischief ensued, it may be presumed that the parents of the children upon whom these experiments were made, would have thought otherwise ; and might have been tempted to take disagreeable and even illegal steps to revenge themselves. Luckily, nothing serious ensued, except that it was then asserted, that vaccine pustules were a common occurrence, and long lists of cases were published where this eruption had taken place.

"The sagacious discoverer had been too attentive to the phenomena of the disease to deserve being suspected of overlooking so obvious a circumstance. He maintained with modesty, that pustular eruptions had never occurred to his patients ; and he was persuaded that some mistake had been committed ; and that perhaps the vaccine fluid employed was not of a proper kind.

"This suggestion gave great offence to those who had published pustular cases ; they were alarmed lest their skill should be questioned and their practice diminished. From interested friends they became insidious foes ; and even endeavoured to frustrate the generous disposition of Parliament in his favour.

"No sooner had abuse commenced than Dr. Jenner with propriety withdrew from the contest. But others warmly espoused his cause. The newspapers became infected with virulent paragraphs ; an eruption of confluent pamphlets broke out ; inflammatory duodecimos succeeded, and swelling octavos full of matter burst from the press. Many ingenious hypotheses were formed to account for the eruptions. Some supposed they were small-pox, others cow-pox, and a third party a hybrid disease. A few minute philosophers, in order to sift this business to the bottom, determined to examine the matter of the pustules by the solar microscope, and by the nicest chemical tests. But in the mean time, Dr. Jenner's rules for inoculation were silently put in practice ; upon which the eruptions suddenly vanished, and no pus could be found to make experiments with.

"Jenner never took advantage of this conclusion to triumph over his adversaries. The source of their error is now pretty well understood, and he allowed this ridiculous dispute to die away. Such was the issue of the teazing controversy betwixt the friends of vaccination ; but we must now attend to a more vehement one with its opponents."

We have offered this as a specimen of the author's lively manner of writing, and also to correct an incacuracy, which though apparently trifling, has, like every other error, been productive of inconveniences. Though the pustules above alluded to, were certainly not vaccine, yet the most accurate observers have found vaccination occasionally attended with secondary eruptions. They are indeed, such as cannot be mistaken for variolous, being different in their form, in their contents, in the time of their appearance, and in not communicating any infection by effluvia. Dr. Jenner, with his usual candour, admits them, and has by inoculating from them, produced the genuine disease.

We thought it right to mention thus much, not that it at all detracts from Dr. Jenner's moderation as commended by Mr. Moore, but because in this, and in all other cases, we conceive truth the first object. Indeed, when we consider the analogy of every other morbid poison with which we are acquainted, it would be a strange peculiarity if this alone should be invariably free from any secondary eruptions. At the same time it may be right to add, that such secondary vesicles are very rare, and when they do appear are unattended with fever, or any other inconvenience.

We shall conclude this article with remarking, that we have perused the whole with much pleasure, and without meeting with any thing objectionable, excepting what we have noticed above. We recommend it as the fittest work on the subject for the higher classes, whilst Dr. Adams's, as we before remarked, is best calculated for those who have fewer advantages of education.

---

*Letters to Dr. Rowley, on his late Pamphlet, entitled 'Cow-pox Inoculation, no Security against Small-pox Infection.'* By Aculeus. Octavo, pp. 60. London, 1805.

This little pamphlet consists of a set of smart letters to Dr. Rowley, with an appendix, in which some well authenticated facts are related.

This is certainly the proper way to answer the absurdities contained in Dr. Rowley's pamphlet. There are performances on which cool reasoning is lost, because they are neither founded on facts, nor supported by any just inferences, even from the statement of the author himself. In these cases, as there is nothing to refute, Horace's adage is very just:

"Ridiculum acri
"Rectius et melius plerumque secat res."

But as the force of wit is much weakened if the whole of the attendant circumstances are not aptly introduced, we shall transcribe an entire letter, as a specimen of the rest. The title page being embellished with a wooden cut of the ox-faced boy, and a cow grazing behind him, we have selected Letter X. to which the subject refers.

"*Most Learned Sir,*

"Had Swedenborg possessed *your* talent for investigating truth, and explaining theological mysteries, the world would have been *illuminated* indeed! *He* pretended to be favoured with celestial visions for the benefit of his fellow-creatures, and, with the dull reasoning of a mathematician, laboured, by voluminous writings, to demonstrate, that his doctrines were conformable to Scripture, and the character of the Supreme Being as a good and wise father of his children; but you, more able Sir, have developed, in *three* lines of text, and *six* of *note*, a mystery hid from the foundation of the world! Your own native genius, and not any borrowed light light from the Angelic Heaven, directs your researches; and, instead of "a *raving mad follower*" of the Swedish divine, you stand confessed as the polar-star of divinity. *You* boast an intimate knowledge of the decrees of Heaven, and shew what is ordained for the visitation of man, and what he has brought upon himself by his presumption. You, sir, demonstrate, *without argument*, the right of inoculation for the small-pox, and clearly prove, *in the same manner*, that to inoculate for the cow-pock, or even the chicken-pox, is an impious and flagrant "violation of our *most holy religion:*" for, who can be so blind as not to see, that whether a man "lies with a beast, and contaminates the *form of the Creator* with the brute creation;" or, whether he dips his lancet in the pellucid virus on a milk-maid's hand, and thus raises a vesicle on the arm of his child; he is alike guilty of *bestiality;* alike obnoxious to the divine law, and all the penalties of human justice?—Thus are the Vaccinators convicted; not only of "a breach of the sixth commandment," as noticed in my former letter; but of actual intercourse with the brute creation!! "The contemplative and *learned* ministers of the Gospel," whom you piously call upon to corroborate your doctrine, cannot fail to perceive, that, altho' *small-pox* inoculation is perfectly allowable to man as a free, rational, and accountable agent; and that all the opposition once made to the practice, as "*wresting out of the hands of the Almighty the divine dispensations of his Providence*," arose from gross ignorance and barbarous, absurd superstition; yet, that this very same objection, when brought against the *cow-pock*, is perfectly rational, religious, and conclusive.—What a degeneracy shall we witness in the next generation, contaminated, deformed, and *brutalized by* "*millions* over the whole face of the earth!" what signal punishment awaits "presumptuous man," for this "*daring violation of our most Holy Religion!!*" If, according to LORD MONBODDO, men once resembled the brute creation by having TAILS; may we not, according to Dr. ROWLEY, expect to find them in turn, graced with HORNS? Will not our grand-sons and grand-daughters forget the human accent, and low responsive to the grazing herds; or, leaving their mother's breast, associate with the beasts of the field? shall we not behold, in addition to the many sheep-faced boys who now trudge daily to school, and the hoggish boors who break the clod, a future race, of mungrel aspect and intellect, more resembling CALVES than human beings; will not the natural consequence be, that, instead of our children sending their sons to study "*Anatomy, Physiology, Pathology,* and *Therapeutics*," we shall see them quartered for sale on the butchers' shambles? Dreadful thought! yet consequences no less shocking are to be expected, if "*Mankind*" continue, in despite of every friendly and *disinterested* admonition, in the "beastly" practice of Vaccination. Be it so.—You, sir, are clear in the sight of God and man;—*your* hands are washed in innocency;—and when divine justice shall sweep with the besom of destruction the impious herd of

 vaccinists vaccinists from the face of the earth, or visit a guilty and polluted land with universal dissolution; *you* at least will escape "unhurt amidst the war of elements, the wreck of matter, and the crush of worlds!"

"I turn from this awful scene, to contemplate the noble candour with which, notwithstanding their crimes, you treat the Vaccinators, in acknowledging their claims to superiority, by admiting their strongest argument in favour of the new practice: but lest this might appear to argue an inconsistency on your part, I shall devote my next letter to a proper explanation of this subject; and reconcile this apparent contradiction, by shewing the real motive which leads to such candour; and I hope to be able fully to prove, that this part of your Treatise is in exact conformity to your main design, namely, the promotion of your "*new, improved, refined, and successful mode of small-pox inoculation.*"

---

*Observations on Vaccine Inoculation, tending to confute the Opinions of Dr. Rowley and others.* By HENRY FRASER, M. D. &c.

THIS is a light performance, neatly written, and containing some handsome compliments to the late Dr. Woodville and to Dr. Jenner. The principal novelty in it is, that the author conceives he has refuted the opinion of the origin of the cow-pox from the grease of the horse. We believe at the time this pamphlet was written, the last volume of Memoirs of the Medical Society was not published. In our next number we shall offer that volume to the notice of our readers.

---

*Remarks on the Report of M. Chaptal (late Minister of the Interior) to the Consuls or former Government of France; with an Examination of the Claim of M. Guiton de Morveau to the Discovery of the Power of the Mineral Acid Gases on Contagion; in a Letter addressed to William Wilberforce, Esq. M. P. &c.* By JAMES CARMICHAEL SMITH, M. D. &c. Octavo. London, 1805.

THERE cannot be a more important subject in Medicine, than the means of purifying infected apartments. The application of sulphuric acid has been known from an earlier period than can be ascertained. But to use this effectually, the chamber must be unihabited as the fumes of that mineral are found fatal to all animals.

It is well known that Dr. Carmichael Smith substituted the fumes of nitrous acid, and so much to the satisfaction of a Committee of the House of Commons, as to receive a reward of five thousand pounds for his discovery.

M. Chaptal disputes the honour of the invention; but by the pamphlet before us, it is evident that his arguments and statements of facts, only go to prove, that M. Morveau had used the muriatic acid acid for the same purposes for which Dr. C. Smith applied the nitrous.

Now it is admitted that a powerful evolution of the muriatic acid is painful and dangerous, particularly to those in reduced health, by the effect it has on the lungs; and if the evolution is sufficiently sudden and considerable, that the consequence may be fatal.

We pretend not to ascertain whether nitrous acid has all the advantages ascribed to it, as this has been disputed by some who have had large opportunities of knowing its effects. However, the weight of evidence appears in its favour; and to the discovery of its safe exhibition in inhabited chambers, we think there cannot be a doubt that Dr. Smith has a fair title.

---

*An Essay on the Diseases incident to Indian Seamen or Lascars on long Voyages.* By WILLIAM HUNTER, A. M. Member of the Asiatic Society of Calcutta, Foreign Member of the Medical Society of London and Paris, and Surgeon to the Company's Marine. Folio, 1804, Calcutta, printed at the Company's press.

THIS is a valuable compilation on a subject of great importance. The author informs us, that being called upon in the execution of his duty as marine surgeon to examine into the cause of an unusual mortality among the native crews of some of the country ships which returned from England in the year 1801, the singularity of the disease, together with its rapid and fatal progress, arrested his attention, and he resolved to lose no opportunity of collecting further information on the subject. In the course of this inquiry, he met with other varieties of disease which he here proceeds to enumerate, with such remarks as the materials in his possession enable him to offer. Those materials principally consist of reports made to the Board of Trade by the master-attendant, and marine-surgeon, or assistant surgeon in charge of the marine.

These various diseases are particularized with some suitable practical remarks on their causes, and the mode of treating and preventing them. On the last the author is most particular, referring for the more minute description and history of the diseases, to the appendices, which consist principally of reports from different master's attendants. Several useful observations are added, and such copious references to the various authors who have written on the diseases, or those which are most analogous to them, as very much to enhance the value of the work.

---

*Vaccinæ*

*Vaccinæ Vindicia*; *or, a Vindication of the Cow-pox: containing a Refutation of the Cases, and Reasonings on the same, in Dr. Rowley's late extraordinary Pamphlet against Vaccination, in a Letter to Dr. Moseley*. By ROBERT JOHN THORNTON, M.D. 8vo. London, 1806.

THIS is only the first part of Dr. Thornton's Answer to Dr. Rowley. After a Dedication to the Jennerian Society, the author announces his intentions with the following letter:

" SIR,

" I HAD hoped my labours were ended, having *proved*, as I thought, the *superior advantages* of the COW-POX, over the SMALL-POX, INOCULATION; when I found to my great astonishment, that two physicians of no small eminence in the profession, who had before often enlightened mankind, and evinced great erudition, and deep practical skill with regard to the science of medicine, entertained sentiments diametrically opposite to these, and tried to support them by a number of *Facts*.—I had indeed looked upon your first sentiments as the product of a *playful* hour, " serious trifling," as you were pleased to call them; but when you again appear in print in 1805, together with Dr. Rowley in the same year, both whose works run through two editions, I see with considerable pain and anxiety, that certain men, however wise in other respects, may, upon *one subject*, display a total want of that clearness of mind and judgment so conspicuous on other occasions; I was about to say that *prejudice* may have so warped the understanding, that we do not indeed recognize the same individuals.

" Dr. Rowley has declared his work to be " a solemn appeal, not to the passions of mankind, but to the reason and judgment of all who are capable of deep reflection; and, it is hoped, the *well-authenticated irrefutable facts* produced, will recieve a cool dispassionate consideration."

" In a work, where fair discusscon should be admitted, and in which strict truth should be the object of free inquiry, it is absolutely necessary to establish *facts* to prevent controversy."

" It is sincerely wished, that the most severe investigations [on cow and small-pox inoculation, may be liberally instituted by all those who are *disinterested*, not by the *ex parte* accounts of vaccinators, and all idle disputation or altercation be suspended; for no *arguments* nor *controversy*, however artfully contrived, however ingeniously constructed, can overturn *visible* and *indubitable facts*."

" Finally, *truth* is the object of this inquiry; to sacred *truth* alone should contending parties make their appeal, and submissively abide by her decision."

" Having invited an examination into " *facts*," which I agree with Dr. Rowley can alone ultimately establish the propositions on either side, I hope in this arduous investigation I shall not lose the liberality and candour of a *gentleman*. To abuse in *retaliation* would would be a sign of weakness on our part, and every voice during such altercation would be crying out to come to *proofs*.

"To be brief, I here solemnly protest I have no other motive but the *public good*, and very reluctantly undertook to expose the errors Dr. Rowley, yourself, and a few others have been under, with regard to vaccination."

This Part contains many pointed observations, and shows how little attention Dr. Rowley paid to accuracy in collecting what he calls facts. When the whole appears before us we shall be more minute in our observations. In the next Part, which is to appear in March, we trust the author will correct an error in the names of the Medical Council of the Jennerian Society. Though there is no doubt, that the Gentlemen of the former Council, hold similar opinions with the present, on the subject of vaccination, yet, a reference to our last Number, would have enabled Dr. T. to have given the signatures to the Report with accuracy.

